# Genetic susceptibility of early aseptic loosening after total hip arthroplasty: the influence of TIMP-1 gene polymorphism on Chinese Han population

**DOI:** 10.1186/s13018-014-0108-1

**Published:** 2014-12-03

**Authors:** Fengyu Pan, Shan Hua, Yi Luo, Dongjun Yin, Zhuang Ma

**Affiliations:** Department of Orthopaedics, Chinese People’s Liberation Army 107 Hospital, No.7 South Zhichu Road, Yantai, 264002 China; Department of Radiology, Yantai Haigang Hospital, Yantai, Shandong China

**Keywords:** Total hip arthroplasty, Genetic factor, Single nucleotide polymorphisms

## Abstract

**Objective:**

Genetic factor plays an important role in early failure of total hip arthroplasty (aseptic loosening) etiology, and TIMP-1 gene may be involved. The present study was conducted to reveal possible association between TIMP-1 polymorphisms with the risk of early failure of total hip arthroplasty (THA) (aseptic loosening).

**Methods:**

The TIMP-1 single nucleotide polymorphisms (SNPs) rs4898, rs6609533, and rs2070584 were genotyped in 59 subjects who were diagnosed as aseptic loosening after total hip arthroplasty and in 100 controls.

**Results:**

The TIMP-1 SNP rs4898 T allele in the case group was found to be 1.32 fold (*P* = 0.0013, 95% CI = 1.16 to 1.58) than the control group. Similarly, the G allele of rs6609533 was found to be associated with increased risk of aseptic loosening (OR = 1.78, 95% CI = 1.52 to 2.17, *P* < 0.0001). For SNP rs2070584, no statistical association was found (A vs. G, OR = 1.14, 95% CI = 0.97 to 1.40, *P* = 0.2028).

**Conclusion:**

The results showed that the TIMP-1 SNPs rs4898 and rs6609533 were associated with the increased risk of early aseptic loosening susceptibility.

## Introduction

There are numerous patients with end stage arthritis treated with cemented total hip arthroplasty (THA), and the number is rapidly increasing [1-4]. Almost 1 million of THA are implanted worldwide annually, with a predicting increase of 174% to nearly 600,000 THA procedures annually by 2030 in the United States [5-7]. However, indispensable proportion of patients after THA still faces the complications that may lead to the premature prosthesis failure and revision surgery, with significant impact on their quality of life [5]. While sepsis, fracture, and dislocation are relatively rare, loosening of the total hip prosthesis arising from aseptic inflammatory reactions is one of the most critical phenomena (accounts for 75.7% of all THA revisions) in the treatment of total hip arthroplasty [8-10].

The aseptic loosening after THA has been considered to be due to both mechanical stress and local host responses to the implanted materials including the wear debris of high density polyethylene, bone cement, and metals; however, the precise biological mechanisms responsible for loosening have not yet been completely clarified [11-14]. Nowadays, it is considered that the prosthesis stimulates the inflammatory response of mesenchymal cells and accumulates the osteoclast, leading to excessive resorption, bone loss, and eventually periprosthetic osteolysis [15,16]. However, given the existence of the individual difference of susceptibility to aseptic loosening, there may be a combination of environmental and genetic factors influencing the pathologies. Environmental factors have been widely studied across the world, including the implant design, material, type of prosthesis, fixation method, surgical technique, and postoperative rehabilitation procedure [17,18]. However, the individual susceptibility difference was mostly related to the genetic factors like single nucleotide polymorphisms (SNPs). Various gene SNPs of GNAS1, TNF-238, TNF-a, IL6-174, MMP1, MMP2, etc. were reported to be associated with increased prosthetic loosening.

The release of implant particles and debris into the periprosthetic tissues leads to the formation of reactive granulation tissue against a foreign body, and it activates the cells to produce cytokines and enzymes [19]. Previous studies demonstrated that this granulomatous tissue has the ability to produce inflammatory cytokines and substances, i.e., interleukin-1 (IL-1), platelet-derived growth factor (PDGF), prostaglandin E2 (PGE2), etc. [20]. Matrix metalloproteinases (MMPs) are a family of enzymes secreted by mesenchymal and haemopoietic cells that can degrade various types of collagen, which is the most important component of the extracellular matrix (ECM). They are active at neutral pH and are all inhibited by specific secreted inhibitors, tissue inhibitors of metalloproteinases (TIMPs). Recently, many studies acknowledged that MMPs play an important role in the variety of tissue destruction, i.e., metastatic tumor, rheumatoid arthritis, and cartilage breakdown [21]. Numerous studies have demonstrated that specific MMPs and TIMPs are expressed in the periprosthetic tissues and are critically involved in the bone resorption and subsequent implant failure [22-27]. SNPs of MMP1 have been related to increased risk of prosthetic loosening. However, no genetic research between TIMPs polymorphism and aseptic loosening after THA has been performed so far. This study aims to determine whether the TIMP-1 SNPs rs4898, rs6609533, and rs2070584 were associated with failure of THA (aseptic loosening) in Chinese Han population.

## Method

The study was approved by the ethics committee of the Chinese People’s Liberation Army 107 Hospital, and informed consent was obtained from patients and control participants.

### Study population

Fifty-nine patients diagnosed with aseptic loosening after total hip arthroplasty at the Department of Orthopaedics of Chinese People’s Liberation Army 107 Hospital were enrolled in this study. All the patients were diagnosed based on the findings of clinical, radiological, laboratory, and intra-surgical changes. The diagnosis criteria of aseptic loosening were documented as follows: 1. Clinical symptoms like hip pain during walking or moving the joint. 2. Radiological changes like migration of prosthetic or bone radiolucency around the prosthesis of more than 2 mm. 3. Abnormal laboratory data like erythrocyte sedimentation rate and leukogram. Patients were excluded if they had any deep infection or the suspicion of implant infection, inflammatory diseases, traumatic loosening, or immunosuppressant agents after THA in their history. Also, aseptic loosening developed after 10 years since THA was excluded from this study. The control group consists of 100 age- and gender-matched patients who had undergone THA that had been seen to be therapeutically successful over long-term follow-up. All subjects included in this study were Chinese Han population.

### Genotyping

DNA samples were obtained from all the participants from peripheral blood with the Chelex 100 method [28]. The primers and probes were designed and synthesized by Sigma (Sigma-Proligo, The Woodlands, TX). The SNPs were genotyped using Taqman assay (Applied Biosystems 7500, ABI, Foster City, CA) and dual-labeled probes in real-time PCR. Genotyping was performed by an independent laboratory personnel, and two authors independently reviewed the results. Disagreements were resolved through a discussion and consensus. In addition, 5% samples of the case and control subjects were randomly selected for reproducibility tests at least twice to yield a 100% concordant.

### Statistical analysis

Statistical Package for Social Sciences software (SPSS Inc., Chicago, IL, USA), version 16.0 for Windows and HaploView software were used for statistical analysis in this study. The demographic and clinical data were presented as mean ± SD and compared between groups by the student’s *t* tests. The genotype and allelic frequencies were evaluated by Hardy–Weinberg equilibrium and compared by the Chi-square test. Multivariate logistic regression was used to estimate odds ratios (ORs) and 95% confidence intervals (CI) after adjustment for age, gender, and body mass index (BMI). The linkage disequilibrium (LD) mapping and the associations between haplotypes of selected SNPs and risk of aseptic loosening after THA were estimated by HaploView software. The *P* < 0.05 was considered to indicate a statistically significant difference.

## Results

### Patient characteristics

Demographic data of the subjects enrolled were shown in Table 1. There were no significant differences between groups in respect to the age, gender, and BMI. The mean period after THA was 8.2 ± 1.5 years when the aseptic loosening happened, and the control group has a mean period of 12.7 ± 2.3 years after THA.

**Table 1:** **The summary of the basic characteristics of the groups**

**Clinical characteristics**	**Aseptic loosening patients**	**Controls**	***P*** **value**
Number	59	100	
Age (years)	68.6 ± 8.4	70.3 ± 7.1	n.s
Female/male	22/37	36/64	n.s
BMI (kg/m^2^)	27.4 ± 5.9	26.9 ± 6.6	n.s
Mean time after THA (years)	8.2 ± 1.5	12.7 ± 2.3	<0.001

### Association of TIMP-1 polymorphisms with susceptibility to failure of THA

As expected, the distribution of the genotypes of SNPs of TIMP-1 gene conformed to the Hardy–Weinberg equilibrium and the genotyping success rate was 100%. Table 2 listed the genotyped and allele distributions of the three SNPs for the cases and controls. The TIMP-1 SNPs rs4898 and rs6609533 were found to be significantly associated with an increased risk of THA failure after adjustment of age, gender, and BMI. The TIMP-1 SNP rs4898 T allele in the case group was found to be 1.32 fold (*P* = 0.0013, 95% CI = 1.16 to 1.58) than the control group. Similarly, the G allele of rs6609533 was found to be associated with an increased risk of aseptic loosening (OR = 1.78, 95% CI = 1.52 to 2.17, *P* < 0.0001). For SNP rs2070584, no statistical association was found (A vs. G, OR = 1.14, 95% CI = 0.97 to 1.40, *P* = 0.2028).

**Table 2:** **The genotype and allele distributions of the three SNPs for the cases and controls**

**Group**	**rs4898 Genotype**			**Allele (%)**			
	**CC**	**CT**	**TT**	**C**	**T**	**OR (95%CI)** ^**a**^	***P*** ^**a**^
Control	36	45	19	58.5	41.5	1.32 (1.16 to 1.58)	0.0013
Case	16	29	14	51.7	48.3		
Group	rs6609533 Genotype			Allele (%)			
	TT	TG	GG	T	G	OR (95%CI)^a^	*P* ^a^
Control	45	37	18	63.5	36.5	1.78 (1.52 to 2.17)	< 0.0001
Case	13	32	14	49.2	50.8		
Group	rs2070584 Genotype			Allele (%)			
	GG	AG	AA	G	A	OR (95%CI)^a^	*P* ^a^
Control	49	40	11	69.0	31.0	1.14 (0.97 to 1.40)	0.2028
Case	27	24	8	66.1	33.9		

^a^ORs and 95% CIs were estimated using multiple logistic regression analyses and adjusted for age, gender, and BMI.

**Figure 1:**
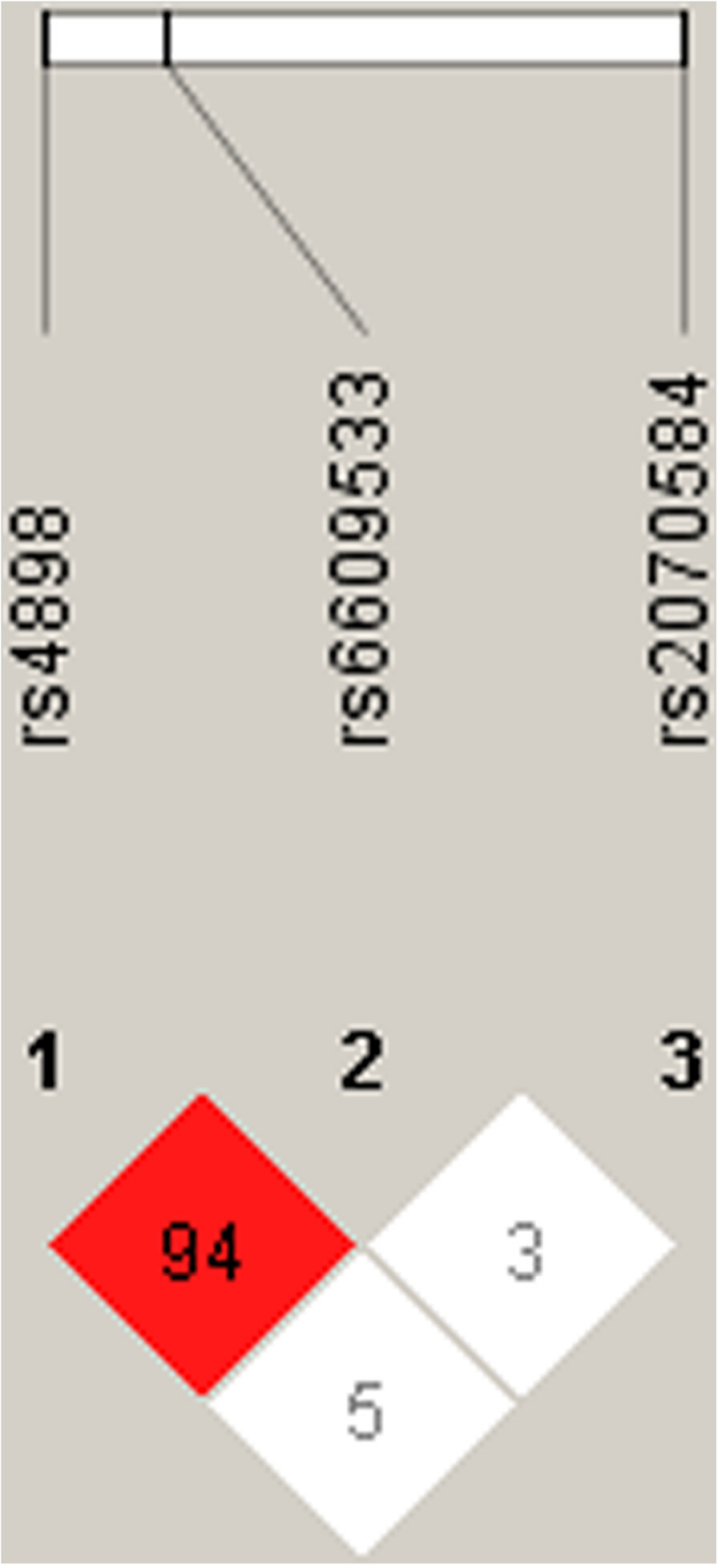
**The LD across the TIMP-1 gene.** The results of LD mapping are generated using Haploview software. The values for D’ between each SNP are presented in each box.

The LD within TIMP-1 gene was only found between rs4898 and rs6609533, showing that these two polymorphisms belong to one haploblock (Figure 1).

## Discussion

This is the first study investigating the possible role of TIMP-1 SNPs in the pathologies of aseptic loosening of the total hip prosthesis. The present study revealed the association between TIMP-1 SNPs rs4898 and rs6609533 with the increased risk of early failure of THA (aseptic loosening).

Although total hip arthroplasty is a mature and successful surgery that improves the quality of life significantly, it cannot be ignored that there are still many patients suffering from early failure of the THA. Importantly, aseptic loosening due to the periprosthetic osteolysis is known to be the main reason for an early failure after THA.

Although many reports have been published on the pathogenesis of periprosthetic loosening, the precise biological mechanisms responsible for this process have not yet been completely elucidated. Biological, microbiological, biomechanical factors, etc. have been considered to introduce the aseptic loosening. However, the abnormal immunological responses caused by the wear-generated particular debris surrounding the implant components were considered as the core of the pathologies [29,30]. The periprosthetic loosening and osteolysis were found to be correlated with higher wear rates of the prosthesis [29]. The experimental systems also demonstrated that particulate debris could induce osteolysis in a variety of animal models and inflammatory responses in cultured macrophages [31,32]. The release of particles induced an immunological response which is taking place in the capsular tissue initially. The macrophages, osteoclasts, fibroblasts, etc. were activated and produced various chemical factors and mediators [17,33-36]. These soluble factors migrate in the interface between the implant and the bone, where they continue the immune response but mainly affected the bone tissues.

ECM degradation and connective tissue remodeling around implants have been considered as major biological events in the periprosthetic loosening. Among which the MMPs and TIMPs play as critical mediators of wear particle-induced inflammatory osteolysis. MMP is one of the cytokines and lipid mediators that increases the destruction of the extracellular organic matrix of bone [22,23,37-39]. The expression of MMP-13 was found in the macrophages, endothelial cells, and fibroblasts of the synovial-like membrane around the implant [35]. Local messenger RNA (mRNA) expression profile also showed that MMP-1, MMP-9, MMP-10, MMP-12, and MMP-13 were strongly elevated in aseptic loosening compared to controls. Furthermore, collagen degradation in periprosthetic tissue correlated significantly with the number of local MMP-1 and MMP-13 [36]. Recently, Malik and coworkers reported the association of a MMP-1 SNP rs5854 and the occurrence of increased aseptic loosening susceptibility [40]. The proteolytic activity of MMPs is regulated by specific TIMPs. The balance between the levels of activated MMPs and free TIMPs determines in part the net MMP activity. In addition to regulating the MMPs, TIMPs have also been shown to have angiogenic and growth factor-like activities [41]. Quantitative analysis of mRNA expression of TIMPs in periprosthetic tissues showed a significant upregulation of TIMP-1, TIMP-2, and TIMP-3 in contrast to the decreased levels of TIMP-4 [25]. The strong expression of TIMP-2 in the interface tissue around implants was also reported by Ishiguro and coworkers [26].

The most important limitation of the present study is the relatively small sample size. A single center case–control study is not sufficient to fully interpret the relationship between TIMP-1 polymorphisms and the risk of early failure of THA (aseptic loosening). Further study with multiple center and larger sample size is needed. Also, our investigation is only a genetic association study, and the precise impact of this polymorphism on protein function has not been confirmed by molecular biology techniques.

## Conclusions

The present study was conducted to reveal possible association between TIMP-1 SNPs with the risk of early failure of THA (aseptic loosening). The results showed that the TIMP-1 SNPs rs4898 and rs6609533 were associated with the increased risk of early aseptic loosening susceptibility.
